# Delayed-onset post-operative keratitis and endophthalmitis caused by *Exophiala oligosperma*

**DOI:** 10.1186/s12348-021-00277-9

**Published:** 2021-12-23

**Authors:** Laurie W. van der Merwe, Dawood da Costa, Kessendri Reddy, David Meyer

**Affiliations:** 1 Division of Ophthalmology, Tygerberg Academic Hospital, University of Stellenbosch, Cape Town, South Africa; 2 Division of Medical Microbiology and Immunology, Department of Pathology, Faculty of Medicine and Health Sciences, Stellenbosch University and National Health Laboratory Service, Tygerberg Hospital, Cape Town, South Africa

**Keywords:** Fungal endophthalmitis, Fungal keratitis, *Exophiala oligosperma*, Delayed-onset endophthalmitis

## Abstract

A case of delayed-onset post-cataract-surgery keratitis and endophthalmitis, caused by the melanin-producing fungus *Exophiala oligosperma,* is presented. The patient presented with an infection at the corneal side-port wound 5 months after an uneventful phacoemulsification surgery. Despite pars plana vitrectomy and combination antifungal treatment, the patient required an evisceration of the globe. Limited clinical information is available about the treatment of eye infections caused by this organism.

## Introduction

Delayed-onset post-operative endophthalmitis can be a frustrating entity to manage. Fungi, a frequent cause of this syndrome, may replicate slowly, can be difficult to culture and often respond poorly to antifungal agents. We present a case of delayed-onset post-operative keratitis and endophthalmitis caused by *Exophiala oligosperma* in a resource-limited population.

## Presentation

A 75-year-old female patient presented to the ophthalmology outpatient clinic at Tygerberg Hospital, South Africa, 1 week after the spontaneous onset of pain and photophobia in her left eye. She was known to have type II diabetes with an HbA1C of 8.2% and ischaemic heart disease. She was HIV-negative and did not use immunosuppressive therapy. The patient underwent an uncomplicated phacoemulsification procedure with an intraocular lens (IOL) implantation of the affected eye 5 months before the onset of her symptoms.

On examination, her right (unaffected) eye had an uncorrected distance visual acuity (VA) of 0.9, a normal pseudophakic anterior and posterior segment examination and no diabetic retinopathy. The VA in the left (affected) eye was counting fingers at 1 m, which improved to Snellen VA of 0.2 with a pinhole. No blepharospasm or chemosis was present. Ciliary injection was noted, with a white corneal stromal infiltrate at the seven o’clock side-port wound, without epithelial staining. The infiltrate was round and less than 1 mm diameter, with surrounding corneal oedema and fine corneal precipitates. The anterior chamber was formed, without the presence of any cells or flare, and no precipitates were visible on the intraocular lens. She did not have a relative afferent pupillary defect (RAPD).

**Fig. 1:**
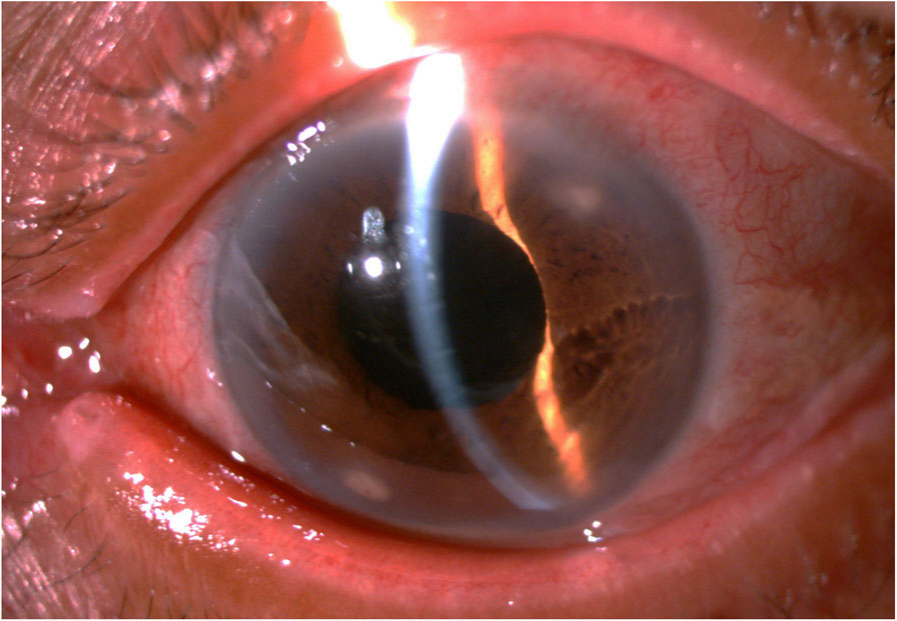
Infiltrate at corneal side-port wounds in left eye

With the diagnosis of keratitis, topical ciprofloxacin 0.3% was prescribed, with frequent follow-up. Although symptoms improved initially, the infiltrate persisted, and a possible immunological process was suspected due to the peripheral intrastromal location and lack of an epithelial defect. The infiltrate was too small and deep to get an adequate sample, and a corneal biopsy was not considered at this stage because the infiltrate still appeared to be benign. In addition to the ciprofloxacin, a topical steroid-antibiotic combination of polymyxin B, neomycin and dexamethasone (Maxitrol®) was prescribed with close clinical follow-up. The patient reported further improvement in her symptoms, but the infiltrate remained unchanged. Three weeks after defaulting on a scheduled appointment, the patient returned to the clinic with enlargement of the infiltrate at the seven o’clock side-port site, a new infiltrate at the two o’clock side-port wound and an anterior-chamber reaction with 1+ flare and 2+ cells. The Maxitrol® was stopped, and the antibiotic prescription was changed to fortified topical ceftazidime 5% and cefazolin 5%. A corneal swab and scrape (sample set 1 - see Table 1) from the seven o’clock side-port wound was taken at the slit lamp and sent for microscopy, culture and sensitivity testing, but no organisms were cultured on this sample. Her condition continued to deteriorate, and she developed a slither of hypopyon and 1+ vitreous cells with a VA of counting fingers at 1 m (see Fig. 1). She was then diagnosed with delayed-onset post-operative endophthalmitis and admitted to hospital.

**Table 1:** Summary of samples taken and culture results

Date	Samples	Culture results
**09/05/2019**	**Sample set 1** – corneal swab and scrape	No growth
**28/6/2019**	**Sample set 2** - Vitreous aspirate and corneal swab, scrape, and intrastromal exudate aspiration	Cultured *Exophiala oligosperma* on the vitreous aspirate
**31/7/2019**	**Sample set 3** – vitreous aspirate, anterior chamber aspirate and cornea swab	Cultured *Exophiala oligosperma* on all 3 samples.
**08/08/2019**	**Sample set 4** - Vitreous aspirate	No organisms cultured

On admission vitreous aspirate and corneal scrape samples (sample set 2) were taken, after which she was treated with intravitreal vancomycin (1 mg in 0.1 ml), ceftazidime (2 mg in 0.1 ml) and dexamethasone (0.4 mg in 0.1 ml). At this stage the corneal infiltrate had progressed to form an intrastromal abscess, which was drained and sent for microscopy, culture and sensitivity testing, together with the vitreous and corneal samples (as part of sample set 2). After administration of the intravitreal drugs, the anterior chamber reaction improved, pain resolved despite persistence of 1+ vitreous cells and her unaided VA improved to 0.4. Ten days after obtaining sample set 2, the fungal and bacterial cultures for both aerobic and anaerobic organisms were confirmed to be negative, and the patient was discharged from hospital. The clinical improvement in symptoms and the negative culture results were interpreted as a favourable response to the intravitreal therapy at this point.

**Fig. 2:**
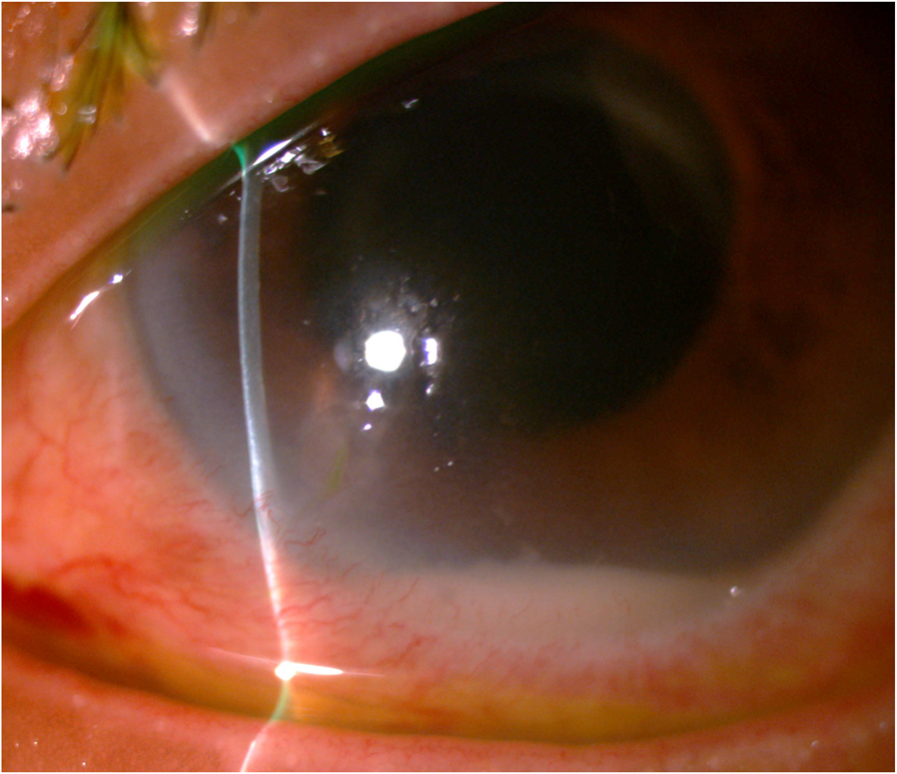
Enlargement of hypopyon and corneal infiltrate in left eye

**Fig. 3:**
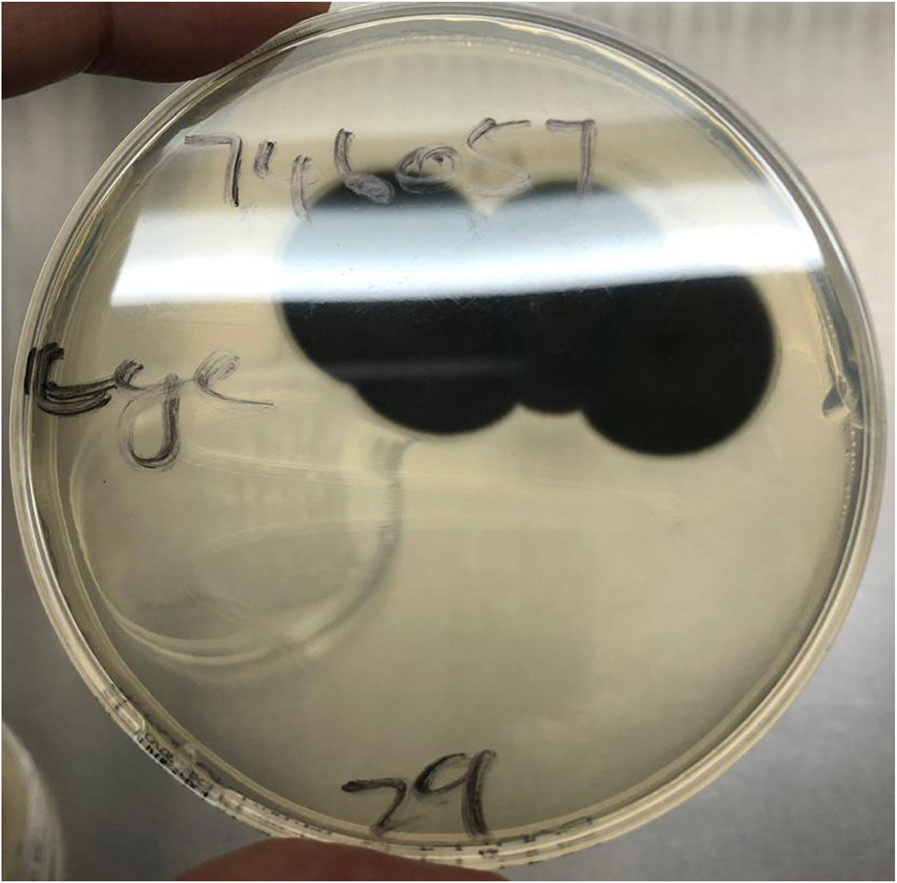
Culture plate of melanin producing Exophiala oligosperma

However, 3 weeks after being discharged, she returned to the clinic with a complaint of recurrent pain and decreased vision in the affected eye. Her vision has deteriorated to detection of hand movements only. A view of the retina was obscured by hypopyon, 4+ cells and 2+ flare, an enlarged temporal corneal infiltrate, corneal oedema and 2+ vitreous cells (Fig. 2). Review of the fungal culture of the vitreous sample (sample set 2) revealed the growth of a filamentous fungus, *Exophiala oligosperma*. This was confirmed with internal transcribed spacer 2 (ITS2) ribosomal DNA sequencing of the cultured fungal isolate (Fig. 3). The patient was readmitted and started on treatment with oral voriconazole 200 mg twice daily, topical amphotericin B 0.15% hourly (later replaced by natamycin 5%) and topical fortified vancomycin 5% and ceftazidime 5% hourly.

Despite therapy, her eye continued to deteriorate. Pars plana vitrectomy (PPV), corneal abscess drainage and an anterior-chamber washout were performed. The IOL and capsular bag were not removed. Intravitreal amphotericin B (5 μg in 0.1 ml), vancomycin and ceftazidime were given intraoperatively. Specimens from the cornea, anterior chamber and vitreous (sample set 3), taken during surgery, were sent for bacterial and fungal microscopy, culture and sensitivity analysis. On the first day post-PPV, the patient had a fibrinous anterior chamber reaction, and on the second day after surgery the hypopyon recurred. During hospitalisation, she received four dosages of intravitreal amphotericin B (5 μg in 0.1 ml), vancomycin and ceftazidime and two dosages of subconjunctival amphotericin B, with a repeat vitreous aspiration for fungal microscopy, culture and sensitivity (sample set 4) at the time of the last intravitreal injection.

**Fig. 4:**
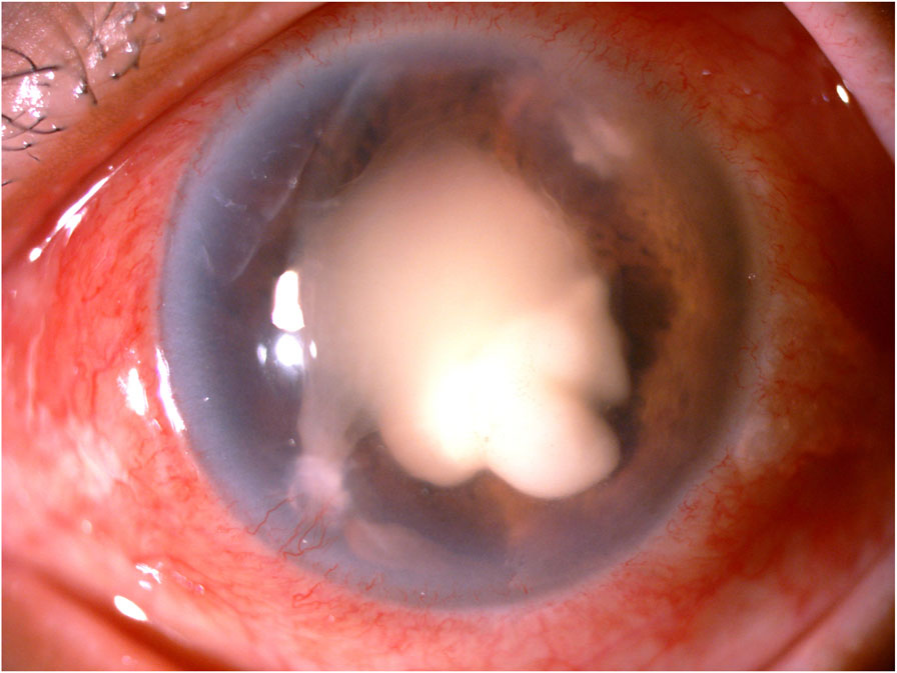
Advanced endophthalmitis prior to evisceration of left eye

Two weeks post-PPV her vision has deteriorated to light perception only. She developed a dense RAPD, recurrence of the intrastromal abscesses at both side-port wounds, a large, localised anterior-chamber exudate collection and persistent pain (Fig. 4). She did not respond to the combination antifungal treatment. All the samples taken during PPV (sample set 3) also cultured *E. oligosperma*. The management options were discussed with the patient, and she opted for an evisceration. The evisceration was performed 8 months after undergoing cataract surgery and 19 days after commencing antifungal treatment.

## Microbiology

Molecular identification was performed on the cultured isolate (Fig. 3). Genomic DNA was extracted using the Quick-DNA Fungal/Bacterial Kit (Zymo Research, USA) according to the manufacturer’s guidelines. A polymerase chain reaction amplification was performed, targeting the internal transcribed spacer region using the ITS2 primers [1]. The obtained fragment sequence was compared with the available sequences in the GenBank using the Basic Local Alignment Search Tool (BLAST) [2]. A BLAST search executed on the sequenced isolate in the National Centre for Biotechnology Information database identified the isolate as *E. oligosperma* with a > 99% homology.

Antifungal susceptibility testing on the cultured isolate of *E. oligosperma* was done at the Mycology Reference Laboratory at the National Institute for Communicable Diseases. The minimum inhibitory concentrations (MIC) were determined by gradient diffusion testing (E test, Biomerieux, USA), and tabulated (Table 1). Clinical breakpoint interpretive criteria do not currently exist for *E. oligosperma.*

## Discussion

Delayed-onset post-operative endophthalmitis is mainly caused by *Propionibacterium* species followed by coagulase-negative staphylococci, *Corynebacterium* species*, Cutibacterium acnes* and fungi [3–9]. Anand et al. reported 21.8% of post-operative endophthalmitis cases in India to be caused by fungi, but the incidence is expected to be lower in the rest of the world [7].

*Exophiala* species are filamentous fungi and form part of a group of dematiaceous moulds. These moulds are darkly pigmented due to the production of melanin in their cell walls, which is also speculated to increase the virulence of these organisms [10, 11]. *Exophiala* species are emerging pathogens known to cause disease in immunocompromised patients, such as people who received transplants, people with HIV and people with diabetes [11, 12]. *Exophiala* species are associated with nutrient-poor and toxic environments. They have been isolated in steam baths and toxic mines [11]. These organisms are known to cause skin and soft tissue infections, pneumonia, keratitis, septic arthritis, endocarditis and neurological infections, with a predilection to form granulomas and microabcesses [11, 12]. It is difficult to distinguish microbiologically between the different species of *Exophiala,* as they show very little morphological differentiation [11, 12]. Chakrabarti et al. ascribed 10.5% of fungal endophthalmitis cases to other melanin-producing fungi, but were not able to culture *Exophiala* species in a single case [13].

Isolated case reports of *Exophiala* infections involving the eye were found in the English literature. Homa et al. conducted a literature review of cases of *Exophiala* eye infections, documented from 1990 to 2018. They reviewed seven cases of keratitis, one case of a subconjunctival mycetoma and seven cases of endophthalmitis [14], and reported on infections caused by *Exophiala dermatitidis, Exophiala jeanselmei* and *Exophiala phaeomuriformis*, but there was no report of an *E. oligosperma* infection. Based on these case reports of *Exophiala* eye infections, pre-existing medical conditions may possibly increase the chances of therapeutic failure. Infections were eradicated in six of the seven cases of keratitis. Conversely, cases of subconjunctival mycetoma and endophthalmitis had poor visual outcomes.

In another case report on *Exophiala phaeomuriformis* keratitis, an elderly Caucasian female with a persistent epithelial defect responded favourably to topical amphotericin B and oral fluconazole [15].

*Exophiala* were not reliably differentiated into species before the advent of widespread molecular testing; as a result, misclassification or under-reporting is plausible. The initial medical management of fungal endophthalmitis consists of antifungal drugs, which may be administered topically, intravitreally or systemically, with up to three different antifungals used simultaneously [5, 8, 16–18].

Oral voriconazole has been found to attain intraocular levels of 1.13 μg/ml in the aqueous and 0.81 μg/ml in the vitreous of non-inflamed eyes [19]. In vitro susceptibility testing has previously shown antifungals to have the following MIC values for *E. oligosperma*: 0.5 μg/ml for voriconazole; 0.25 μg/ml for itraconazole; 0.25 μg/ml for amphotericin B; 0.25 μg/ml for caspofungin and 0.25 μg/ml for natamycin [16].

The antifungal MIC levels were presumably achieved in our patient, and a better response to the combination treatment was expected. Factors that possibly contributed to the poor response are the initial administration of steroid therapy; the primary site of infection being limited to the cornea; the patient’s underlying diabetes and the unknown effect of ocular inflammation on the MIC levels of the antifungal medication. The vitreous sample taken before evisceration (sample set 4) did, however, not culture any fungi.

The topical steroid therapy and single dose of intravitreal dexamethasone might have contributed to the poor outcome. The diagnosis of a fungal keratitis was not initially considered likely because the first corneal swab (sample set 1) did not culture any organisms and there were no clinical signs indicative of a fungal infection. The length of time involved in culturing the fungus significantly hampered the process of diagnosing delayed-onset endophthalmitis.

The prognosis for post-operative fungal endophthalmitis is notoriously poor. Chakrabarti et al. found that 52% of a sample of 53 patients with this diagnosis had a final outcome of counting fingers or worse [13]. In another report, only two from seven cases of endophthalmitis secondary to *Exophiala* infection had improved VA after treatment (voriconazole, fluconazole and surgical intervention for both); the remaining five cases all had unfavourable outcomes [14]. Homa et al. argued that prompt, targeted surgical removal of the infected tissue or IOL improved outcomes; this could be ascribed to the tendency of *Exophiala* species to form localised abscesses. It is suggested that intravitreal voriconazole has better outcomes than intravitreal amphotericin B [14]. Unfortunately we did not have injectable voriconazole available in our hospital at the time, hence the use of amphotericin B.

## Conclusion

This case illustrates that *E. oligosperma* can be pathogenic intraocularly and could embark on a slow but destructive course. The organism is slow to culture, difficult to treat and the use of steroids should be avoided. A high index of suspicion for a fungal keratitis should be entertained in an incisional wound infiltrate, and an early biopsy should be considered, especially with an atypical presentation. Aggressive antifungal therapy with early surgical intervention should be instituted when this fungus is suspected.

## Data Availability

Not applicable.

## Abbreviations

IOL: Intraocular lens
MIC: Minimum inhibitory concentrations
VA: Visual acuity
PPV: Pars plana vitrectomy
RAPD: Relative afferent pupil defect
BLAST: Basic local alignment search tool
MIC: Minimum inhibitory concentrations
